# Early hepatitis B surface antigen decline predicts treatment response to entecavir in patients with chronic hepatitis B

**DOI:** 10.1038/srep42879

**Published:** 2017-02-21

**Authors:** Cheng-Yuan Peng, Hsueh-Chou Lai, Wen-Pang Su, Chia-Hsin Lin, Po-Heng Chuang, Sheng-Hung Chen, Ching-Hsiang Chen

**Affiliations:** 1 School of Medicine, China Medical University, Taichung, Taiwan; 2 Division of Hepatogastroenterology, Department of Internal Medicine, China Medical University Hospital, Taichung, Taiwan; 3 School of Chinese Medicine, China Medical University, Taichung, Taiwan

## Abstract

Early declines in serum hepatitis B surface (HBsAg) levels, their optimal cutoffs, and association with therapeutic endpoints in chronic hepatitis B (CHB) patients receiving entecavir treatment remain unclear. We prospectively enrolled 529 patients (195 hepatitis B e antigen [HBeAg]-positive and 334 HBeAg-negative) with a median treatment duration of 49.2 months. Median HBsAg levels declined significantly in both groups at Month 3, but only at Months 6–12 in the HBeAg-negative group. Both groups exhibited a significant HBsAg decline with each successive year of treatment. An HBsAg decline of ≥75% from baseline, assessed at Months 3 and 12 of treatment in the HBeAg-positive and -negative patients, respectively, independently predicted a virological response and HBeAg seroconversion in the HBeAg-positive patients, an HBsAg level of <100 IU/mL in the HBeAg-negative patients, and HBsAg loss in all the patients during treatment. HBsAg levels of <3,000 IU/mL at baseline combined with an HBsAg decline of ≥75% from baseline provided a predictive algorithm for HBsAg loss (positive and negative predictive values: 70% and 100%, respectively) during 5 years of treatment. The proposed cutoffs for defining an HBsAg decline may assist clinicians in early assessments of treatment responses in genotype B-infected or C-infected CHB patients receiving entecavir therapy.

Hepatitis B surface antigen (HBsAg) assay is a standard serological test for diagnosing hepatitis B virus (HBV) infection. Quantitative serum HBsAg levels correlate with the transcriptional activity of intrahepatic covalently closed circular DNA (cccDNA) of HBV in patients with hepatitis B e antigen (HBeAg)-positive chronic hepatitis B (CHB), but not in those with HBeAg-negative CHB^1^. A decline in HBsAg correlates with a decrease in the intrahepatic total HBV DNA and cccDNA and HBeAg seroconversion (SC) in HBeAg-positive patients receiving peginterferon treatment^2^,^3^. The absolute HBsAg level and HBsAg decline from baseline to Month 3 or 6 of peginterferon treatment can predict the treatment response in HBeAg-positive and -negative patients, respectively^4^,^5^.

HBsAg decline is less pronounced during nucleos(t)ide analog (NA) treatment than during peginterferon treatment^6^. Rapid HBsAg decline, defined as ≥0.5 log_10_ IU/mL at Month 6 or ≥1.0 log_10_ IU/mL at Month 12 of telbivudine treatment, and an HBsAg decline of ≥1.0 log_10_ IU/mL at Month 6 of tenofovir treatment, are associated with subsequent HBsAg loss in HBeAg-positive patients^7^,^8^. However, several crucial concerns remain unresolved. First, the early kinetics of HBsAg levels during NA treatment is not well characterized. Second, the optimal cutoffs and time points for defining HBsAg decline during NA treatment are unclear. Third, the association between early HBsAg decline and the therapeutic outcomes during long-term NA treatment is controversial.

The present study delineated the kinetics of HBsAg levels; identified the optimal cutoffs and time points for defining HBsAg decline; and investigated the association between early HBsAg decline and key therapeutic endpoints such as virological response (VR), HBeAg SC, and HBsAg loss in a large cohort of genotype B-infected or C-infected CHB patients receiving entecavir (ETV) treatment.

## Results

### Baseline characteristics

We enrolled 550 consecutive NA-naïve patients with CHB. Among them, 21 patients were excluded because of missing HBsAg data. Thus, we included 529 patients (195 positive and 334 negative for HBeAg) in the present analysis. The baseline characteristics of all the patients are presented in Table 1. HBeAg-positive patients were significantly younger and showed significantly lower proportions of genotype B infection and cirrhosis; significantly higher platelet counts; higher ALT, HBV DNA, and HBsAg levels; and lower creatinine levels than did the HBeAg-negative patients.

### Treatment outcomes

Median treatment durations for the HBeAg-positive and -negative patients were 49.0 (41.8) and 51.3 (36.4) months, respectively. In total, 169 (86.7%), 142 (72.8%), 102 (52.3%), and 79 (40.5%) HBeAg-positive patients and 289 (86.5%), 229 (68.6%), 173 (51.8%), and 128 (38.3%) HBeAg-negative patients received ETV treatment for more than 2, 3, 4, and 5 years, respectively. The median time to VR was 6 (6) and 3 (3) months in the HBeAg-positive and –negative patients, respectively. The cumulative rates of VR in the HBeAg-positive patients were 77%, 92%, 91%, and 98% at 1, 2, 3, and 4 years, respectively; the corresponding rates in the HBeAg-negative patients were 97%, 99%, 99%, and 99% (Supplementary Fig. S1). The cumulative rates of HBeAg SC in the HBeAg-positive patients were 10.8%, 18.2%, 26.0%, and 32.8% at 1, 2, 3, and 4 years of treatment, respectively (Supplementary Fig. S1). Fourteen (7 HBeAg-positive and 7 HBeAg-negative) patients showed HBsAg loss during a median treatment duration of 33.8 (40.7) months.

### Kinetics of on-treatment serum HBsAg levels

**Figure 1:**
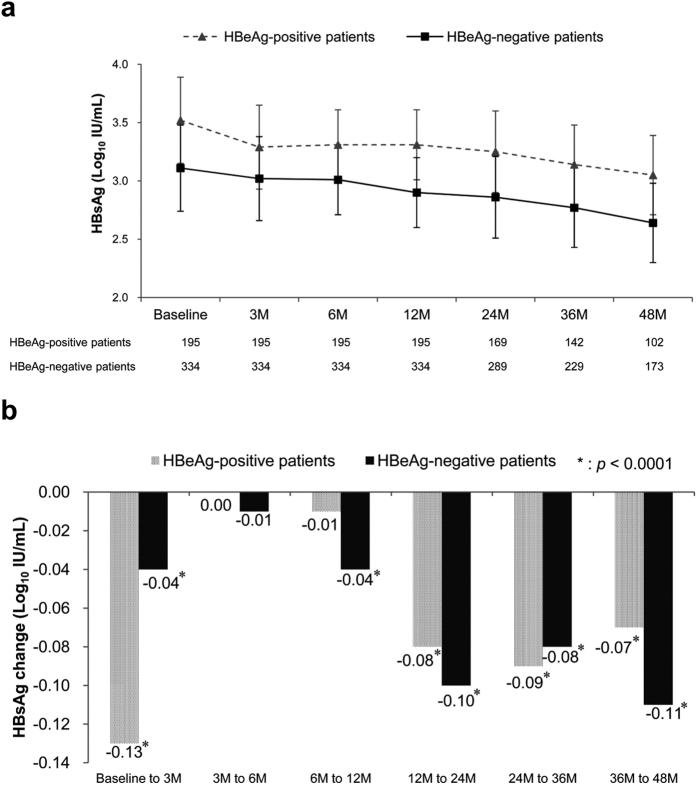
Kinetics of on-treatment serum HBsAg levels. (**a**) Kinetics of serum HBsAg levels during 4 years of entecavir treatment among HBeAg-positive and -negative patients. (**b**) Changes in median HBsAg levels during each successive period of treatment. Error bars indicate the interquartile range. M, months.

We determined the kinetics of the serum HBsAg levels during 4 years of ETV treatment. In the HBeAg-positive patients, the median HBsAg levels declined significantly from baseline to Month 3 of treatment (*p* < 0.0001), but not during Months 3–6 or Months 6–12 of treatment (Fig. 1a and b). In the HBeAg-negative patients, the median HBsAg levels declined significantly from baseline to Month 3 (*p* < 0.0001) and during Months 6–12 of treatment (*p* < 0.0001), but not during Months 3–6 of treatment (Fig. 1a and b). This unique pattern of HBsAg decline during the first year of treatment was consistent in patients who had received ETV treatment for more than 1–4 years (data not shown). HBsAg levels continued to significantly decline in both HBeAg-positive and -negative patients during each successive treatment year (all *p* < 0.0001 during Months 12–24, 24–36, and 36–48 of treatment, Fig. 1b).

### Determination of cutoffs for HBsAg decline

**Figure 2:**
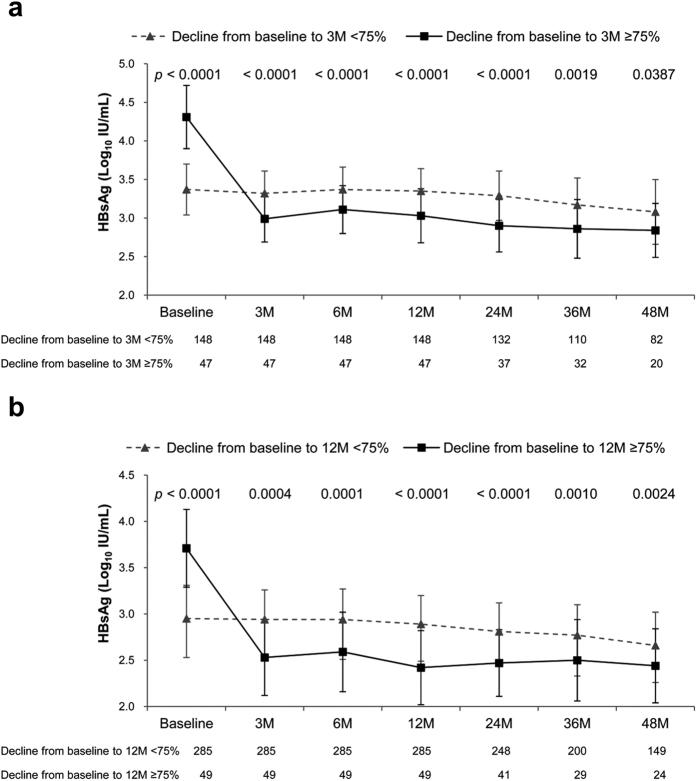
Kinetics of on-treatment serum HBsAg levels stratified by an HBsAg decline of 75% from baseline. (**a**) At 3 months of treatment among HBeAg-positive patients (n = 195) and (**b**) At 12 months of treatment among HBeAg-negative patients (n = 334). Error bars indicate the interquartile range. M, months.

HBsAg levels significantly declined during only the initial 3 months in the first year in HBeAg-positive patients. Therefore, we investigated whether an optimal cutoff point occurred in HBsAg decline at Month 3 of treatment and whether it identified patients with subsequently lower HBsAg levels during treatment than those who did not achieve such an early decline. We plotted the on-treatment kinetics of median serum HBsAg levels by using various arbitrary cutoffs between 10% and 90% to dichotomize patients. For simplicity, only cutoffs of 25%, 50%, 75%, 0.5 log_10_ IU/mL (68.4%), and 1.0 log_10_ IU/mL (90%) are presented herein. The value 0.5 log_10_ IU/mL was selected because of its frequent adoption for studying HBsAg kinetics. Patients who exhibited an HBsAg decline of ≥75% from baseline to Month 3 of treatment achieved a significantly lower HBsAg level at Month 6 and during 1–4 years of treatment than did those who did not exhibit such a decline at Month 3 of treatment (Fig. 2a). The difference was most statistically significant with a cutoff of 75% (Fig. 2a, Supplementary Fig. S2).

HBsAg levels significantly declined at Month 3 and during Months 6–12 of treatment during the first year in HBeAg-negative patients. Therefore, we investigated whether an optimal cutoff point occurred in HBsAg decline at Month 12 of treatment and whether it identified patients who would subsequently achieve a lower HBsAg level during treatment than those who did not achieve such an early decline. Thus, we conducted analyses similar to those for HBeAg-positive patients. Patients who exhibited an HBsAg decline of ≥75% or 1.0 log_10_ IU/mL from baseline to Month 12 of treatment achieved a significantly lower HBsAg level at Months 3 and 6 of treatment and during 1–4 years than did those who did not exhibit such a decline at Month 12 of treatment (Fig. 2b, Supplementary Fig. S3). The statistical significance of the difference was similar among both cutoffs (Fig. 2b, Supplementary Fig. S3). However, a cutoff of 75% was preferred because it included a higher number of patients (n = 49 versus n = 28). Patients who exhibited an HBsAg decline of ≥75% at Month 3 of treatment and those who showed the same decline at Month 12 of treatment achieved similar median HBsAg levels at 1–4 years of treatment (all *p* > 0.05), which were significantly lower than those in patients who did not exhibit such a decline at Month 12 of treatment (all *p* < 0.05, Supplementary Fig. S4).

Based on the aforementioned analyses, we propose that a cutoff of ≥75% decline in HBsAg levels from baseline to Months 3 and 12 of treatment in HBeAg-positive and -negative patients, respectively, be considered the threshold associated with the subsequent achievement of a lower HBsAg level during treatment.

### HBsAg decline as a predictor of VR and HBeAg SC during ETV treatment in HBeAg-positive patients

We investigated whether an HBsAg decline of ≥75% from baseline to Month 3 of treatment is a predictor of VR at 1 year of treatment in HBeAg-positive patients. Univariate logistic regression analysis identified cirrhosis, low creatinine levels, and HBV DNA and HBsAg levels as the significantly associated factors. An HBsAg decline of ≥75% at Month 3 of treatment was a marginally significant factor (*p* = 0.0777, Supplementary Table S1). Multivariate logistic regression analysis indicated HBsAg levels (odds ratio [OR] 0.238, 95% confidence interval [CI] 0.119–0.474, *p* < 0.0001) and an HBsAg decline of ≥75% at Month 3 of treatment (OR 3.581, 95% CI 1.204–10.655, *p* = 0.0218) as the independent predictors (Supplementary Table S1). A similar statistical analysis of HBeAg-negative patients was not feasible because of their high VR rate (92%) at 1 year of ETV treatment.

We investigated whether an HBsAg decline of ≥75% from baseline to Month 3 of treatment is a predictor of HBeAg SC during ETV treatment. Univariate Cox regression analysis identified alanine aminotransferase (ALT) levels of ≥5× upper limit of normal (ULN), genotype C, and an HBsAg decline of ≥75% at Month 3 of treatment as the significantly associated factors (Supplementary Table S2). Multivariate Cox regression analysis indicated genotype C (hazard ratio [HR] 1.964, 95% CI 1.181–3.267, *p* = 0.0093) and an HBsAg decline of ≥75% at Month 3 of treatment (HR 2.110, 95% CI 1.235–3.605, *p* = 0.0063) as the independent predictors (Supplementary Table S2).

### HBsAg kinetics in relation to the achievement of VR

**Figure 3:**
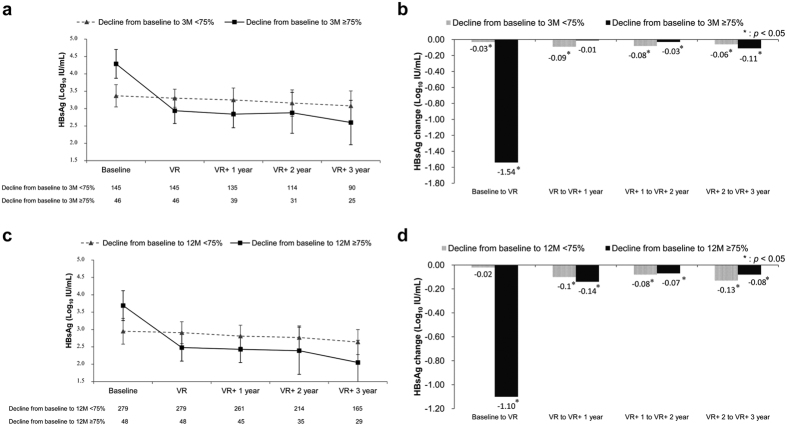
Kinetics of on-treatment serum HBsAg levels in relation to the achievement of VR. (**a**) Stratified by an HBsAg decline of 75% from baseline to Month 3 of treatment in HBeAg-positive patients (n = 191) and (**b**) Changes in median HBsAg levels during each successive period of treatment. (**c**) Stratified by an HBsAg decline of 75% from baseline to Month 12 of treatment in HBeAg-negative patients (n = 327) and (**d**) Changes in median HBsAg levels during each successive period of treatment. Error bars indicate the interquartile range. M, months; VR, virological response.

We investigated the kinetics of the serum HBsAg levels in relation to the achievement of VR among those with or without an early HBsAg decline, defined as ≥75% from baseline to Months 3 and 12 of treatment in HBeAg-positive and -negative patients, respectively. HBsAg levels significantly declined in both HBeAg-positive and -negative patients from baseline to the time of VR and during each successive treatment year following the achievement of VR (all *p* < 0.0001, Supplementary Fig. S5). HBeAg-positive patients with an early HBsAg decline exhibited significant HBsAg decline during each successive treatment period except the first year following VR (Fig. 3a and b). HBeAg-negative patients with an early HBsAg decline exhibited significant HBsAg decline during each successive treatment period (Fig. 3c and d). Furthermore, HBsAg levels significantly declined in both HBeAg-positive and -negative patients without an early HBsAg decline during each successive treatment year following VR (Fig. 3). There were no significant differences in HBsAg decline during each successive treatment year following VR between the patients who exhibited an early HBsAg decline and those who did not in either HBeAg-positive or –negative groups.

### HBsAg decline as a predictor of HBsAg loss during ETV treatment

HBsAg loss is the most desirable therapeutic endpoint of the current NA treatment. We investigated whether early HBsAg decline is a predictor of HBsAg loss. Univariate Cox regression analysis identified cirrhosis, ALT levels of ≥5 × ULN, higher prothrombin time (PT), and an HBsAg decline of ≥75% at Month 3 of treatment in HBeAg-positive patients and at Month 12 of treatment in HBeAg-negative patients as the significantly associated factors (Table 2). Multivariate Cox regression analysis showed HBsAg levels of < 3,000 IU/mL (HR 10.324, 95% CI 2.805–37.992, *p* = 0.0004) and an HBsAg decline of ≥75% (HR 5.820, 95% CI 1.679–20.177, *p* = 0.0055) as the independent predictors of HBsAg loss during treatment (Table 2).

We compared the HBsAg declines before and after the achievement of VR during treatment between the patients who lost HBsAg (n = 14) and those who did not (n = 504). Patients who lost HBsAg exhibited a significantly greater median HBsAg decline both from baseline to the time of VR (1.52 versus 0.07 log_10_ IU/mL, *p* < 0.0001) and annually thereafter (0.50 versus 0.09 log_10_ IU/mL/year, *p* = 0.0008) compared with those who did not.

### HBsAg decline as a predictor of an HBsAg level of <100 IU/mL during ETV treatment in HBeAg-negative patients

HBsAg loss is a rare event during long-term NA treatment; therefore, we used an alternative therapeutic endpoint, an HBsAg level of <100 IU/mL, as preliminary evidence demonstrated its association with the durability of NA treatment in HBeAg-negative patients^9^,^10^. After excluding 28 HBeAg-negative patients who had exhibited a baseline HBsAg level of <100 IU/mL, 57 of 306 HBeAg-negative (18.6%) patients achieved an HBsAg level of <100 IU/mL during treatment. We investigated whether early HBsAg decline is a predictor of ultimately achieving an HBsAg level <100 IU/mL. Univariate Cox regression analysis revealed ALT levels of ≥5 × ULN, HBsAg levels of <3,000 IU/mL, and an HBsAg decline of ≥75% at Month 12 of treatment as the significantly associated factors (Table 3). Moreover, multivariate Cox regression analysis identified HBsAg levels of <3,000 IU/mL (HR 5.604, 95% CI 2.416–12.999, *p* < 0.0001) and an HBsAg decline of ≥75% at Month 12 of treatment (HR 8.238, 95% CI 3.806–17.828, *p* < 0.0001) as the independent predictors of an HBsAg level of <100 IU/mL during treatment (Table 3).

### Confirmation of a threshold of ≥75% as the optimal cutoff for defining an HBsAg decline during ETV treatment

To further confirm that a threshold of ≥75% was the optimal cutoff for defining an HBsAg decline during ETV treatment, we evaluated whether various cutoffs (25%, 50%, 75%, 0.5 log_10_ IU/mL, and 1.0 log_10_ IU/mL) can serve as independent predictors of key therapeutic endpoints such as VR, HBeAg SC in HBeAg-positive patients, an HBsAg level <100 IU/mL in HBeAg-negative patients, and HBsAg loss in all patients after adjustment for baseline confounders. A decline of ≥75% was the only cutoff that predicted all these key endpoints, thus confirming its optimal clinical relevance (Supplementary Table S3).

### Predictive values of an HBsAg decline for therapeutic endpoints in patients with CHB receiving ETV treatment

**Figure 4:**
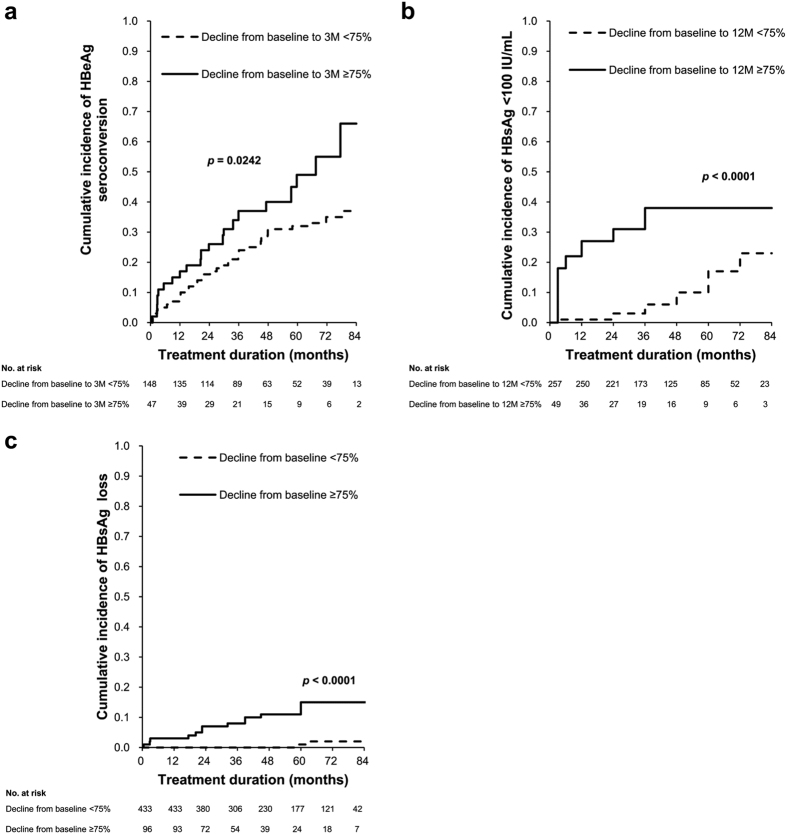
Cumulative incidence of key therapeutic endpoints. Cumulative incidence of (**a**) HBeAg seroconversion in HBeAg-positive patients (*p* = 0.0242), (**b**) HBsAg <100 IU/mL in HBeAg-negative patients (*p* < 0.0001), and (**c**) HBsAg loss in all patients (*p* < 0.0001) stratified by an HBsAg decline of 75% from baseline. All values were obtained using the log-rank test. M, months.

Kaplan–Meier analyses revealed that the cumulative incidences of HBeAg SC were significantly different between HBeAg-positive patients with and without an HBsAg decline of ≥75% at Month 3 of treatment (*p* = 0.0242, Fig. 4a) and those of <100 IU/mL HBsAg were significantly different between HBeAg-negative patients with and without an HBsAg decline of ≥75% at Month 12 of treatment (*p* < 0.0001, Fig. 4b). In addition, Kaplan–Meier analysis revealed that the cumulative incidences of HBsAg loss were significantly different between patients with and without an HBsAg decline of ≥75% (at Months 3 and 12 of treatment for HBeAg-positive and -negative patients, respectively; *p* < 0.0001, Fig. 4c).

**Figure 5:**
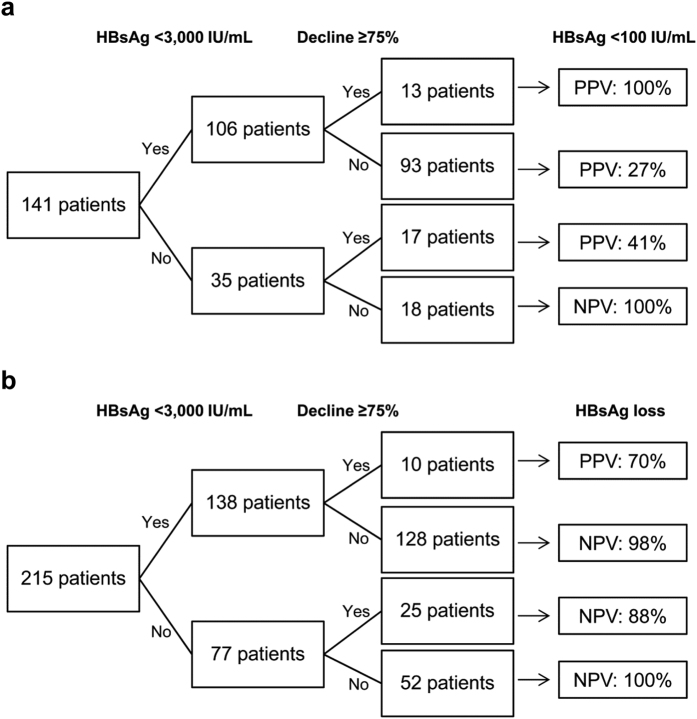
Predictive values of the algorithms based on baseline HBsAg levels and an HBsAg decline of ≥75% from baseline. Algorithms for achieving (**a**) an HBsAg level of <100 IU/mL and (**b**) HBsAg loss during 5 years of entecavir treatment.

Baseline HBsAg levels combined with an HBsAg decline of ≥75% provided a simple algorithm for early prediction of the ultimate achievement of a low HBsAg level during 5 years of ETV treatment. HBeAg-negative patients with baseline HBsAg levels of <3,000 IU/mL and an HBsAg decline of ≥75% exhibited a positive predictive value (PPV) of 100% for <100 IU/mL HBsAg during 5 years of treatment, whereas patients without the two characteristics exhibited a negative predictive value (NPV) of 100% for <100 IU/mL HBsAg (Fig. 5a). Patients with baseline HBsAg levels of <3,000 IU/mL and an HBsAg decline of ≥75% exhibited a PPV of 70% for HBsAg loss during 5 years of treatment, whereas those without either characteristic exhibited an NPV of 88–100% for HBsAg loss (Fig. 5b).

## Discussion

Over 4 years of ETV treatment, HBsAg levels declined at a median annual rate of 0.12 (0.29) and 0.09 (0.16) log_10_ IU/mL in the HBeAg-positive and -negative patients, respectively. The decline was most pronounced during the initial 3 months of treatment in the first year. HBeAg-negative patients exhibited further significant HBsAg decline during Months 6–12 of treatment. An HBsAg decline of ≥75% from baseline was achieved in 24.1% of HBeAg-positive patients at Month 3 of treatment and in 14.7% of HBeAg-negative patients at Month 12 of treatment. An HBsAg decline of ≥75% from baseline, assessed at Months 3 and 12 of treatment in HBeAg-positive and -negative patients, respectively, was identified as an independent predictor of VR and HBeAg SC in HBeAg-positive patients, an HBsAg level of <100 IU/mL in HBeAg-negative patients, and HBsAg loss in all patients during long-term treatment. Baseline HBsAg levels combined with an HBsAg decline of ≥75% provided a simple algorithm for early prediction of the ultimate achievement of a low HBsAg level during 5 years of ETV treatment.

Our results are consistent with those of a previous study, which reported that the overall HBsAg decline was less pronounced in patients receiving ETV treatment compared with those receiving interferon treatment^6^. However, we further demonstrated that the highest decline during the first year of treatment occurred within the initial 3 months, particularly in HBeAg-positive patients. HBsAg levels continued to significantly decline with each successive year of treatment^11^,^12^,^13^,^14^. HBsAg levels significantly declined from baseline to the time of VR and during each successive treatment year following VR.

Until date, no consensus has been reached on the optimal cutoff and time point for defining an HBsAg decline of predictive relevance during NA treatment. Studies have reported an HBsAg decline of ≥1.0 log_10_ IU/mL at Months 12 and 6 of telbivudine and tenofovir treatments, respectively, as a criterion for identifying patients having an increased possibility of HBsAg loss over 3 and 5 years of treatment, respectively^7^,^8^. It is unclear how the cutoffs and time points were derived in these studies. The present proposal was based on four key observations. First, HBsAg levels significantly declined at Month 3 of treatment in both HBeAg-positive and -negative patients and during Months 6–12 of treatment in HBeAg-negative patients. Second, systematic statistical analyses of associations between the magnitude of HBsAg decline and absolute HBsAg levels achieved during 4 years of treatment demonstrated that an HBsAg decline of ≥75% from baseline was most significantly associated with the subsequent achievement of a lower HBsAg level during treatment. Third, multivariate logistic and Cox regression analyses revealed its association with VR, HBeAg SC, and <100 IU/mL HBsAg as well as HBsAg loss during long-term treatment. Fourth, algorithms incorporating HBsAg levels at baseline and an HBsAg decline of ≥75% exhibited favorable predictive capability for ultimately achieving a low HBsAg level during 5 years of ETV treatment. Therefore, this early measurement of HBsAg decline may assist clinicians in determining the possibility of key therapeutic endpoints during a later course of NA treatment.

Our results may explain why some previous studies have neither found an association between HBsAg decline defined by arbitrary cutoffs and key therapeutic endpoints nor underscored the clinical relevance of early HBsAg decline during ETV treatment^6^,^12^,^15^,^16^,^17^,^18^,^19^,^20^. In addition, our results highlight the prerequisite for a low baseline HBsAg level and early HBsAg decline of a sufficient magnitude for ultimately achieving a low HBsAg level, which is currently considered related to the durability of NA treatment in HBeAg-negative patients^9^,^10^. HBsAg loss is associated with continued HBsAg decline both before and after the achievement of VR, which is different from the result of a previous study^21^.

The relatively low number of patients who achieved HBsAg loss in this study may reduce its statistical significance. The mechanisms underlying an early significant HBsAg decline during NA treatment require elucidation^21^. The proposed cutoff requires validation in an independent large cohort for establishing its feasibility as a guide for conducting early assessments in patients with CHB receiving ETV treatment. Whether the same cutoff can be used for predicting a response early during NA treatment in patients with genotype A or D infection and in those receiving different NA treatments warrants further investigation.

In conclusion, we propose a new cutoff for defining an HBsAg decline at specific time points after determining the cutoff through an unbiased statistical analysis and demonstrating its association with key therapeutic endpoints in patients with CHB receiving ETV treatment. Adopting the proposed cutoff may assist clinicians in early assessments of treatment responses.

## Materials and Methods

### Patients

We prospectively enrolled consecutive NA-naïve CHB patients who received ETV treatment (0.5 mg daily) with indications according to the guidelines of the Asian Pacific Association for the Study of the Liver at our unit from June 2006 to June 2014^22^. The inclusion criteria were an age of ≥18 years, the presence of serum HBsAg for at least 6 months, and ETV treatment for >12 months. The exclusion criteria were liver diseases caused by other etiologies, decompensated cirrhosis or hepatocellular carcinoma at baseline, comorbid diseases or cancer, and the concurrent use of immunomodulatory agents.

The study was conducted in accordance with the Helsinki Declaration of 1975. All patients provided written informed consent before enrollment. The study was approved by the Research Ethics Committee of China Medical University Hospital, in Taichung, Taiwan (CMUH102-REC1-113).

### Laboratory examinations

Complete blood cell count, PT, and serum levels of albumin, ALT, total bilirubin, and creatinine were measured at baseline. During ETV treatment, serum ALT levels were measured every 3 months and more frequently when clinically indicated. HBeAg and anti-HBe antibodies (Architect i2000 assay; Abbott Diagnostics, Abbott Park, IL, USA) were detected every 3 months during treatment in the HBeAg-positive patients. HBsAg levels were quantified retrospectively in the patients enrolled before September 2009 and prospectively in those enrolled thereafter by using Abbott Architect HBsAg QT assays (dynamic range, 0.05–250 IU/mL) at baseline; 3, 6, and 12 months; and annually thereafter. Serum HBV DNA levels were measured at baseline; 3, 6, and 12 months; and every 6 months thereafter. The COBAS Amplicor HBV monitor test (lower limit of detection, 50 IU/mL; Roche Diagnostic Systems, Branchburg, NJ, USA) and COBAS AmpliPrep-COBAS TaqMan HBV test (lower limit of detection, 12 IU/mL; Roche Diagnostic Systems) were used before and after August 2008, respectively. HBV genotyping was performed as previously described^23^. Liver fibrosis (F) was staged as no fibrosis (F0), portal fibrosis without septa (F1), portal fibrosis with a few septa (F2), numerous septa without cirrhosis (F3), and cirrhosis (F4) according to the METAVIR system^24^. Cirrhosis was defined on the basis of either 1) histology or 2) repeated ultrasonographic findings suggesting cirrhosis in addition to clinical features such as splenomegaly, thrombocytopenia, ascites, or gastroesophageal varices.

### Therapeutic endpoints

VR was defined as a serum HBV DNA level of <50 IU/mL during ETV treatment. HBeAg SC was defined as the absence of serum HBeAg and presence of anti-HBe antibodies in the HBeAg-positive patients. We assessed the achievement of an HBsAg level of <100 IU/mL and loss of HBsAg, defined as the absence of serum HBsAg.

### Statistical analyses

HBsAg and HBV DNA across a range of 10^2^–10^5^ IU/mL and 10^5^–10^8^ IU/mL, respectively, are presented in logarithmic units where appropriate (2.0 log_10_ = 100; 2.5 log_10_ = 316; 3.0 log_10_ = 1,000; 3.5 log_10_ = 3,162; 4.0 log_10_ = 10,000; 4.5 log_10_ = 31,623; 5.0 log_10_ = 100,000; 5.5 log_10_ = 316,228; 6.0 log_10_ = 1,000,000; 6.5 log_10_ = 3,162,278; 7.0 log_10_ = 10,000,000; 7.5 log_10_ = 31,622,777; 8.0 log_10_ = 100,000,000). Continuous variables, presented as the median (interquartile range), were compared between two groups by using the Mann–Whitney U test. Categorical variables were analyzed using the chi-squared or Fisher exact test, as appropriate. The Wilcoxon signed-rank test was used to investigate changes in HBsAg levels at two time points. Logistic and Cox regression analyses were used to identify factors associated with VR and HBeAg SC, <100 IU/mL HBsAg, or HBsAg loss, respectively. Receiver operating characteristic curves were used to determine the optimal cutoffs that maximize (sensitivity + specificity) for HBsAg levels. Kaplan–Meier analysis and the log-rank test were used to compare the cumulative rates of <100 IU/mL HBsAg and HBsAg loss among the patient subgroups. SAS Version 9.4 (SAS Institute, Inc., Cary, NC, USA) was used for all statistical analyses. A two-sided *p* value of <0.05 was considered significant.

## Additional Information

**How to cite this article**: Peng, C.-Y. *et al*. Early hepatitis B surface antigen decline predicts treatment response to entecavir in patients with chronic hepatitis B. *Sci. Rep.* **7**, 42879; doi: 10.1038/srep42879 (2017).

**Publisher's note:** Springer Nature remains neutral with regard to jurisdictional claims in published maps and institutional affiliations.

## Supplementary Material

Supplementary Information

Supplementary Information with Changes

## Figures and Tables

**Table 1:** Baseline patient characteristics.

Variables	Total	HBeAg-positive	HBeAg-negative	*p*
Median (IQR) or n (%)	(n = 529)	(n = 195)	(n = 334)	
Age: years	49 (16)	42 (13)	53 (15)	<0.0001
Sex (male)	387 (73.2)	142 (72.8)	245 (73.4)	0.8939
Biopsy number	277 (52.4)	103 (52.3)	174 (52.1)	
Fibrosis stage				0.1013
F0/F1	51 (18.4)	25 (24.3)	26 (14.9)	
F2	85 (30.7)	32 (31.1)	53 (30.5)	
F3	35 (12.6)	8 (7.8)	27 (15.5)	
F4	106 (38.3)	38 (36.9)	68 (39.1)	
Cirrhosis (yes)^a^	194 (36.7)	60 (30.8)	134 (40.1)	0.0313
Albumin: g/dL	4.1 (0.6)	4.1 (0.6)	4.1 (0.7)	0.9485
ALT: IU/L	87 (241)	111 (575)	78.5 (166)	0.0003
Total bilirubin: mg/dL	1.06 (0.8)	1.09 (1.14)	1.06 (0.68)	0.2618
PT: seconds prolonged	1.33 (2.02)	1.4 (2.31)	1.2 (1.97)	0.1541
Platelet: ×10^3^/μL	155 (76)	166 (71)	148.5 (72)	0.0029
Creatinine: mg/dL	0.86 (0.27)	0.85 (0.27)	0.86 (0.25)	0.0330
Genotype				<0.0001
B	327 (63.0)	98 (50.5)	229 (70.5)	
C	192 (37.0)	96 (49.5)	96 (29.5)	
HBV DNA: log_10_ IU/mL	5.8 (2.69)	7.04 (2.47)	5.28 (2.29)	<0.0001
HBV DNA: IU/mL	630,957 (490)	10,964,782 (295)	195,046 (195)	<0.0001
HBsAg: log_10_ IU/mL	3.2 (0.81)	3.52 (1.00)	3.03 (0.77)	<0.0001
HBsAg: IU/mL	1,585 (6.6)	3,311 (10)	1,072 (5.9)	<0.0001

ALT, alanine aminotransferase; HBeAg, hepatitis B e antigen; HBsAg, hepatitis B surface antigen; HBV, hepatitis B virus; IQR, interquartile range; PT, prothrombin time.

^a^Cirrhosis diagnosed based on clinical findings or biopsy.

**Table 2:** Univariate and multivariate Cox regression analyses of factors associated with HBsAg loss in patients during entecavir treatment (n = 529).

Variables	Univariate analysis		Multivariate analysis	
	Hazard ratio (95% CI)	*p*	Hazard ratio (95% CI)	*p*
Age: years	0.969 (0.926–1.013)	0.1688		
Sex: male vs female	0.628 (0.210–1.873)	0.4038		
HBeAg: negative vs positive	1.691 (0.593–4.82)	0.3260		
Cirrhosis: no vs yes	0.131 (0.017–0.999)	0.0499		
Albumin: g/dL	1.149 (0.436–3.028)	0.7782		
ALT: ≥5× vs <5× ULN	15.813 (3.535–70.727)	0.0003		
Total bilirubin: mg/dL	1.069 (0.984–1.162)	0.1135		
PT: seconds prolonged	1.167 (1.001–1.361)	0.0484		
Platelet: ×10^3^/μL	1.002 (0.994–1.011)	0.6045		
Cr: mg/dL	0.208 (0.012–3.462)	0.2737		
Genotype: C vs B	0.762 (0.235–2.476)	0.6517		
HBV DNA: log_10_ IU/mL	0.849 (0.630–1.144)	0.2811		
HBsAg: <3,000 vs ≥3,000 IU/mL	2.787 (0.720–10.789)	0.1379	10.324 (2.805–37.992)	0.0004
HBsAg decline: ≥75% vs <75%^a^	9.946 (3.324–29.762)	<0.0001	5.820 (1.679–20.177)	0.0055

ALT, alanine aminotransferase; HBeAg, hepatitis B e antigen; HBsAg, hepatitis B surface antigen; HBV, hepatitis B virus; ULN, upper limit of normal; vs, versus.

Fourteen patients achieved HBsAg loss during entecavir treatment.

^a^An HBsAg decline of ≥75% at the first 3 months of treatment in the HBeAg-positive patients and at the first 12 months of treatment in the HBeAg-negative patients.

**Table 3:** Multivariate Cox regression analyses of factors associated with the achievement of HBsAg <100 IU/mL in HBeAg-negative patients during entecavir treatment (n = 306).

Variables	Univariate analysis		Multivariate analysis	
	Hazard ratio (95% CI)	*p*	Hazard ratio (95% CI)	*p*
Age: years	0.995 (0.971–1.020)	0.6925		
Sex: male vs female	1.105 (0.601–2.033)	0.7471		
Cirrhosis: no vs yes	1.080 (0.628–1.858)	0.7811		
Albumin: g/dL	0.808 (0.519–1.258)	0.3449		
ALT: ≥5× vs <5× ULN	2.526 (1.450–4.400)	0.0011		
Total bilirubin: mg/dL	1.022 (0.963–1.085)	0.4649		
PT: seconds prolonged	1.072 (0.950–1.210)	0.2599		
Platelet: ×10^3^/μL	0.997 (0.992–1.002)	0.2252		
Cr: mg/dL	0.629 (0.192–2.065)	0.4447		
Genotype: C vs B	0.717 (0.408–1.262)	0.2490		
HBV DNA: log_10_ IU/mL	1.078 (0.916–1.268)	0.3655		
HBsAg: <3,000 vs ≥3,000 IU/mL	2.353 (1.097–5.045)	0.0279	5.604 (2.416–12.999)	<0.0001
HBsAg decline at 12 months: ≥75% vs <75%	3.403 (1.908–6.068)	<0.0001	8.238 (3.806–17.828)	<0.0001

ALT, alanine aminotransferase; HBeAg, hepatitis B e antigen; HBsAg, hepatitis B surface antigen; HBV, hepatitis B virus; PT, prothrombin time; vs, versus.

Fifty-seven patients achieved HBsAg <100 IU/mL.

## References

[b1] Thompson A. J. . Serum hepatitis B surface antigen and hepatitis B e antigen titers: disease phase influences correlation with viral load and intrahepatic hepatitis B virus markers. Hepatology 51, 1933–1944, doi: 10.1002/hep.23571 (2010). 20512987

[b2] Wursthorn K. . Peginterferon alpha-2b plus adefovir induce strong cccDNA decline and HBsAg reduction in patients with chronic hepatitis B. Hepatology 44, 675–684, doi: 10.1002/hep.21282 (2006). 16941693

[b3] Chan H. L. . Serum hepatitis B surface antigen quantitation can reflect hepatitis B virus in the liver and predict treatment response. Clin Gastroenterol Hepatol 5, 1462–1468, doi: 10.1016/j.cgh.2007.09.005 (2007). 18054753

[b4] Sonneveld M. J. . Response-guided peginterferon therapy in hepatitis B e antigen-positive chronic hepatitis B using serum hepatitis B surface antigen levels. Hepatology 58, 872–880, doi: 10.1002/hep.26436 (2013). 23553752

[b5] Rijckborst V. . Early on-treatment prediction of response to peginterferon alfa-2a for HBeAg-negative chronic hepatitis B using HBsAg and HBV DNA levels. Hepatology 52, 454–461, doi: 10.1002/hep.23722 (2010). 20683945

[b6] Reijnders J. G. . Kinetics of hepatitis B surface antigen differ between treatment with peginterferon and entecavir. J Hepatol 54, 449–454, doi: 10.1016/j.jhep.2010.07.046 (2011). 21112655

[b7] Wursthorn K. . Kinetics of hepatitis B surface antigen decline during 3 years of telbivudine treatment in hepatitis B e antigen-positive patients. Hepatology 52, 1611–1620, doi: 10.1002/hep.23905 (2010). 20931556

[b8] Marcellin P. . Kinetics of hepatitis B surface antigen loss in patients with HBeAg-positive chronic hepatitis B treated with tenofovir disoproxil fumarate. J Hepatol 61, 1228–1237, doi: 10.1016/j.jhep.2014.07.019 (2014). 25046847 PMC5976831

[b9] Chan H. L. . Prediction of off-treatment response to lamivudine by serum hepatitis B surface antigen quantification in hepatitis B e antigen-negative patients. Antivir Ther 16, 1249–1257, doi: 10.3851/IMP1921 (2011). 22155906

[b10] Chen C. H. . Association between level of hepatitis B surface antigen and relapse after entecavir therapy for chronic hepatitis B virus infection. Clin Gastroenterol Hepatol 13, 1984–1992, e1981, doi: 10.1016/j.cgh.2015.06.002 (2015). 26073492

[b11] Zoutendijk R., Hansen B. E., van Vuuren A. J., Boucher C. A. & Janssen H. L. Serum HBsAg decline during long-term potent nucleos(t)ide analogue therapy for chronic hepatitis B and prediction of HBsAg loss. J Infect Dis 204, 415–418, doi: 10.1093/infdis/jir282 (2011). 21742840

[b12] Chen E. Q. . Quantitative hepatitis B surface antigen titres in Chinese chronic hepatitis B patients over 4 years of entecavir treatment. Antivir Ther 18, 955–965, doi: 10.3851/IMP2579 (2013). 23639885

[b13] Hara T. . Long-term entecavir therapy results in falls in serum hepatitis B surface antigen levels and seroclearance in nucleos(t)ide-naive chronic hepatitis B patients. J Viral Hepat 21, 802–808, doi: 10.1111/jvh.12211 (2014). 25274427

[b14] Seto W. K. . Changes of HBsAg and HBV DNA levels in Chinese chronic hepatitis B patients after 5 years of entecavir treatment. J Gastroenterol Hepatol 29, 1028–1034, doi: 10.1111/jgh.12476 (2014). 24325451

[b15] Gish R. G. . Quantitative hepatitis B surface antigen analysis in hepatitis B e antigen-positive nucleoside-naive patients treated with entecavir. Antivir Ther 18, 691–698, doi: 10.3851/IMP2559 (2013). 23510982

[b16] Zoulim F. . Quantification of HBsAg in nucleos(t)ide-naive patients treated for chronic hepatitis B with entecavir with or without tenofovir in the BE-LOW study. J Hepatol 62, 56–63, doi: 10.1016/j.jhep.2014.08.031 (2015). 25176615

[b17] Jung Y. K. . Change in serum hepatitis B surface antigen level and its clinical significance in treatment-naive, hepatitis B e antigen-positive patients receiving entecavir. J Clin Gastroenterol 44, 653–657, doi: 10.1097/MCG.0b013e3181d52946 (2010). 20216430

[b18] Lee J. M. . Quantitative hepatitis B surface antigen and hepatitis B e antigen titers in prediction of treatment response to entecavir. Hepatology 53, 1486–1493, doi: 10.1002/hep.24221 (2011). 21520167

[b19] Fung J. . Quantitative hepatitis B surface antigen levels in patients with chronic hepatitis B after 2 years of entecavir treatment. Am J Gastroenterol 106, 1766–1773, doi: 10.1038/ajg.2011.253 (2011). 21826112

[b20] Shin J. W. . Prediction of response to entecavir therapy in patients with HBeAg-positive chronic hepatitis B based on on-treatment HBsAg, HBeAg and HBV DNA levels. J Viral Hepat 19, 724–731, doi: 10.1111/j.1365-2893.2012.01599.x (2012). 22967104

[b21] Jaroszewicz J. . Hepatitis B surface antigen (HBsAg) decrease and serum interferon-inducible protein-10 levels as predictive markers for HBsAg loss during treatment with nucleoside/nucleotide analogues. Antivir Ther 16, 915–924, doi: 10.3851/IMP1866 (2011). 21900724

[b22] Liaw Y. F. . Asian-Pacific consensus statement on the management of chronic hepatitis B: a 2012 update. Hepatol Int 6, 531–561, doi: 10.1007/s12072-012-9365-4 (2012). 26201469

[b23] Peng C. Y. . Predictors for early HBeAg loss during lamivudine therapy in HBeAg-positive chronic hepatitis B patients with acute exacerbation. Hepatol Int 5, 586–596, doi: 10.1007/s12072-010-9227-x (2011). PMC3034004 21442057

[b24] The French METAVIR Cooperative Study Group. Intraobserver and interobserver variations in liver biopsy interpretation in patients with chronic hepatitis C. Hepatology 20, 15–20, doi: 10.1002/hep.1840200104 (1994). 8020885

