# Supplementary material for: Early hepatitis B surface antigen decline predicts treatment response to entecavir in patients with chronic hepatitis B
Source: Sci Rep. 2017 Feb 21;7:42879. doi: 10.1038/srep42879 (PMC5318891; doi:10.1038/srep42879)

Supplementary Information

Early hepatitis B surface antigen decline predicts treatment response to entecavir in patients with chronic hepatitis B

Cheng-Yuan Peng, Hsueh-Chou Lai, Wen-Pang Su, Chia-Hsin Lin, Po-Heng Chuang, Sheng-Hung Chen & Ching-Hsiang Chen

Table S1. Univariate and multivariate logistic regression analyses of factors associated with VR at 1 year of entecavir treatment in HBeAg-positive patients (n = 195).

ALT, alanine aminotransferase; HBeAg, hepatitis B e antigen; HBsAg, hepatitis B surface antigen; HBV, hepatitis B virus; PT, prothrombin time; ULN, upper limit of normal; VR, virological response; vs, versus.

A total of 150 patients achieved VR at 1 year of treatment.

Variables	Univariate analysis		Multivariate analysis	
	Odds ratio (95% CI)	*p*	Odds ratio (95% CI)	*p*
Age: years	0.993 (0.964–1.023)	0.6494		
Sex: male vs female	0.624 (0.276–1.412)	0.2577		
Cirrhosis: no vs yes	0.412 (0.178–0.954)	0.0385		
Albumin: g/dL	0.843 (0.457–1.553)	0.5833		
ALT: ≥5× vs <5× ULN	1.765 (0.854–3.645)	0.1249		
Total bilirubin: mg/dL	1.042 (0.926–1.172)	0.4930		
PT: seconds prolonged	1.006 (0.880–1.149)	0.9329		
Platelet: $\times 10^3/\mu L$	0.997 (0.992–1.003)	0.3198		
Creatinine: mg/dL	0.102 (0.013–0.790)	0.0289		
Genotype: B vs C	0.792 (0.405–1.550)	0.4959		
HBV DNA: $\log_{10}$ IU/mL	0.610 (0.474–0.786)	0.0001		
HBsAg: $\log_{10}$ IU/mL	0.351 (0.210–0.587)	< 0.0001	0.238 (0.119–0.474)	< 0.0001
HBsAg decline at 3 months: ≥75% vs <75%	2.365 (0.909–6.154)	0.0777	3.581 (1.204–10.655)	0.0218

Table S2. Univariate and multivariate Cox regression analyses of factors associated with HBeAg seroconversion in HBeAg-positive patients (n = 195).

ALT, alanine aminotransferase; HBeAg, hepatitis B e antigen; HBsAg, hepatitis B surface antigen; HBV, hepatitis B virus; PT, prothrombin time; ULN, upper limit of normal; vs, versus.

A total of 65 patients achieved HBeAg seroconversion during entecavir treatment**.**

**Variables**	**Univariate analysis**		**Multivariate analysis**	
	Hazard ratio (95% CI)	*p*	Hazard ratio (95% CI)	*p*
Age: years	0.993 (0.971–1.016)	0.5448		
Sex: male vs female	0.959 (0.551–1.668)	0.8817		
Cirrhosis: yes vs no	0.991 (0.580–1.692)	0.9732		
Albumin: g/dL	0.688 (0.442–1.070)	0.0968		
ALT: ≥5× vs <5× ULN	2.284 (1.402–3.722)	0.0009		
Total bilirubin: mg/dL	1.020 (0.962–1.081)	0.5112		
PT: seconds prolonged	1.003 (0.909–1.107)	0.9452		
Platelet: <150 vs ≥150 $\times 10^3/\mu L$	1.500 (0.913–2.464)	0.1096		
Creatinine: mg/dL	1.021 (0.537–1.943)	0.9492		
Genotype: C vs B	1.714 (1.044–2.816)	0.0333	1.964 (1.181–3.267)	0.0093
HBV DNA: $\log_{10}$ IU/mL	1.053 (0.915–1.211)	0.4691		
HBsAg: $\log_{10}$ IU/mL	0.877 (0.659–1.168)	0.3699		
HBsAg decline at 3 months: ≥75% vs <75%	1.807 (1.072–3.044)	0.0263	2.110 (1.235–3.605)	0.0063

Table S3. Various cutoffs for HBsAg decline as an independent predictor of key therapeutic endpoints.

HBeAg, hepatitis B e antigen; HBsAg, hepatitis B surface antigen; SC, seroconversion; VR, virological response.

[a]OR: multivariate logistic regression analysis for VR. HR: multivariate Cox regression analyses for HBeAg SC, HBsAg <100 IU/ml, and HBsAg loss.

[b]Adjusted for baseline HBsAg levels.

[c]Adjusted for genotype and baseline HBsAg levels.

**Cutoff**	**25%**		**50%**		**75%**		**0.5 $\log_{10}$ IU/mL**		**1.0 $\log_{10}$ IU/mL**	
	OR/HR[a] (95% CI)	*p*	OR/HR[a] (95% CI)	*p*	OR/HR[a] (95% CI)	*p*	OR/HR[a] (95% CI)	*p*	OR/HR[a] (95% CI)	*p*
**HBeAg–Positive**										
VR[b]	1.503 (0.659–3.431)	0.3331	2.118 (0.902–4.976)	0.0850	3.581 (1.204–10.655)	0.0218	2.919 (1.113–7.652)	0.0294	1.896 (0.611–5.884)	0.2683
HBeAg SC[C]	1.174 (0.712–1.935)	0.5291	1.377 (0.817–2.318)	0.2293	2.110 (1.235–3.605)	0.0063	1.692 (0.980–2.921)	0.0591	2.095 (1.041–4.217)	0.0382
**HBeAg–Negative**										
HBsAg										
<100 IU/mL[b]	2.654 (1.483–4.752)	0.0010	4.002 (2.114–7.576)	< 0.0001	8.238 (3.806–17.828)	< 0.0001	6.223 (3.080–12.573)	< 0.0001	19.854 (7.262–54.280)	< 0.0001
**Total Patients**										
HBsAg loss[b]	6.277 (1.172–23.014)	0.0056	6.139 (1.657-22.744)	0.0066	5.820 (1.679–20.177)	0.0055	13.301 (4.251–41.616)	< 0.0001	32.532 (10.023–105.58)	0.0335

Supplementary Figures

Figure S1. Cumulative rates of key therapeutic endpoints. Cumulative rates of (a) undetectable serum HBV DNA levels in the HBeAg-positive and -negative patients and (b) HBeAg seroconversion in the HBeAg-positive patients.

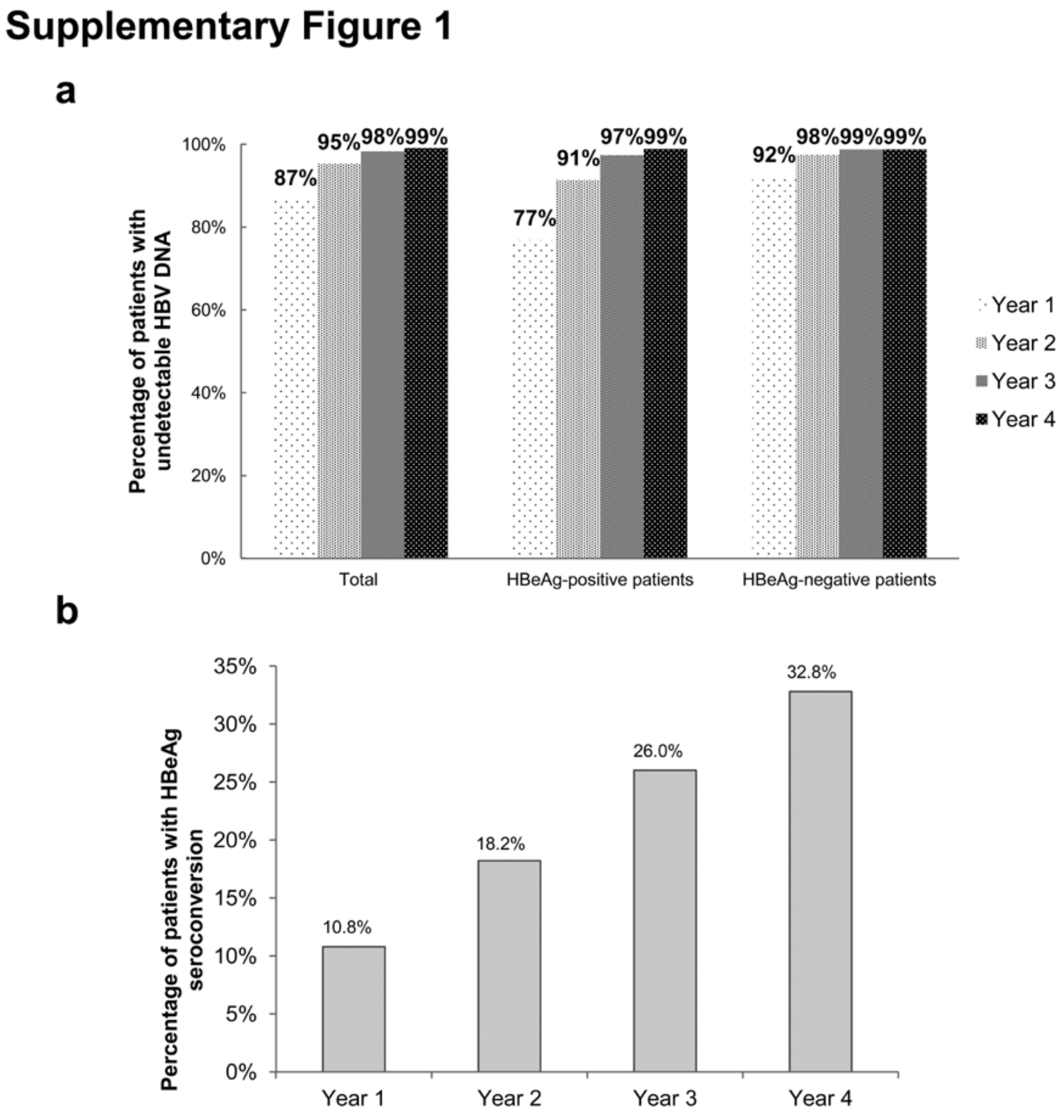

Figure S2. Kinetics of on-treatment serum HBsAg levels, stratified by various cutoffs for an HBsAg decline from baseline, at Month 3 of treatment in the HBeAg-positive patients (n = 195). (a) 25%, (b) 50%, (c) 0.5 log_{10} IU/mL, and (d) 1.0 log_{10} IU/mL. Error bars indicate the interquartile range. M, months.

(a)

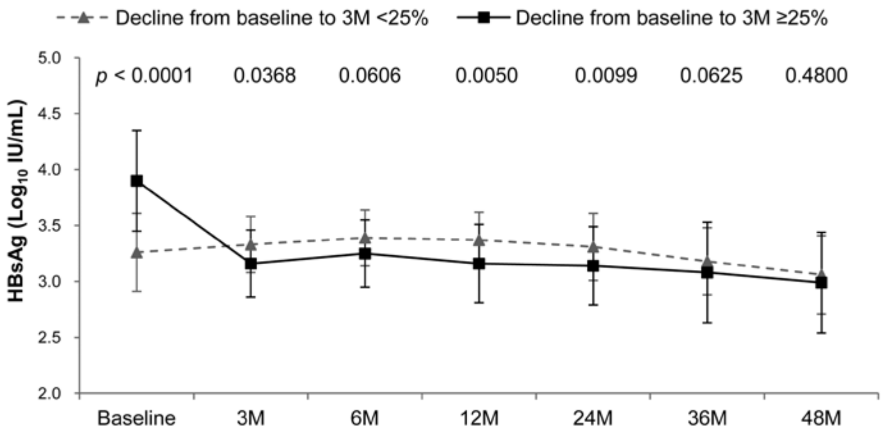

Decline from baseline to 3M <25%	96	96	96	96	86	70	51
Decline from baseline to 3M ≥25%	99	99	99	99	83	72	51

(b)

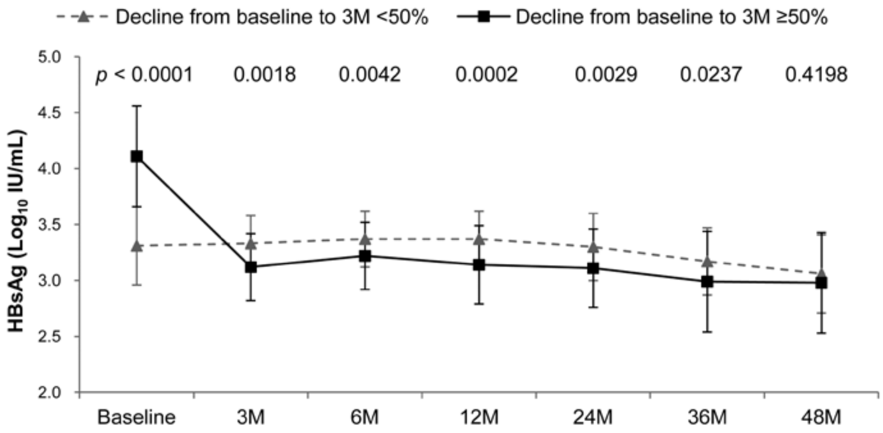

Decline from baseline to 3M <50%	126	126	126	126	110	92	68
Decline from baseline to 3M ≥50%	69	69	69	69	59	50	34

(c)

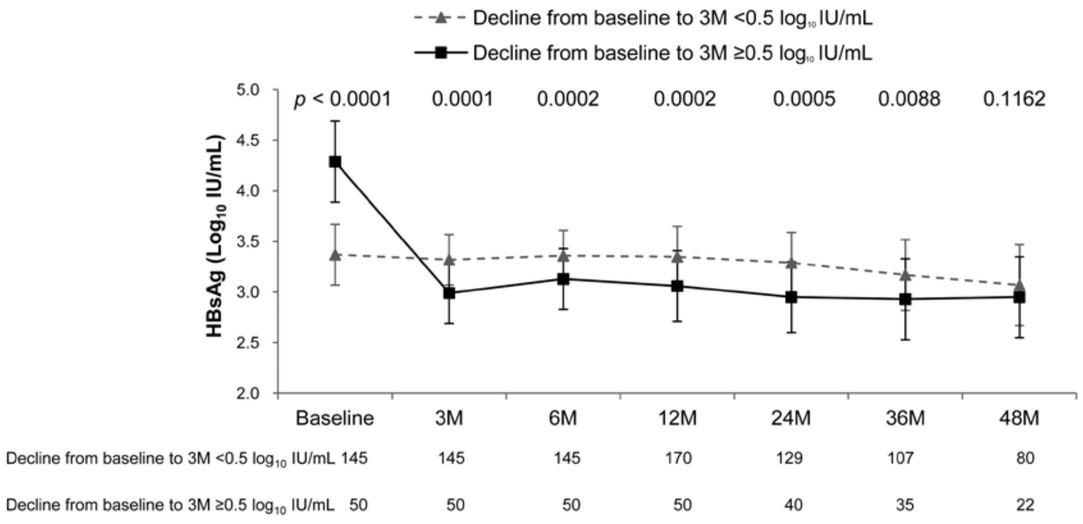
Decline from baseline to 3M <0.5 log10 IU/mL
Decline from baseline to 3M ≥0.5 log10 IU/mL
p < 0.0001
0.0001
0.0002
0.0002
0.0005
0.0088
0.1162
HBsAg (Log10 IU/mL)
5.0
4.5
4.0
3.5
3.0
2.5
2.0
Baseline
3M
6M
12M
24M
36M
48M
Decline from baseline to 3M <0.5 log10 IU/mL 145 145 145 170 129 107 80
Decline from baseline to 3M ≥0.5 log10 IU/mL 50 50 50 50 40 35 22

(d)

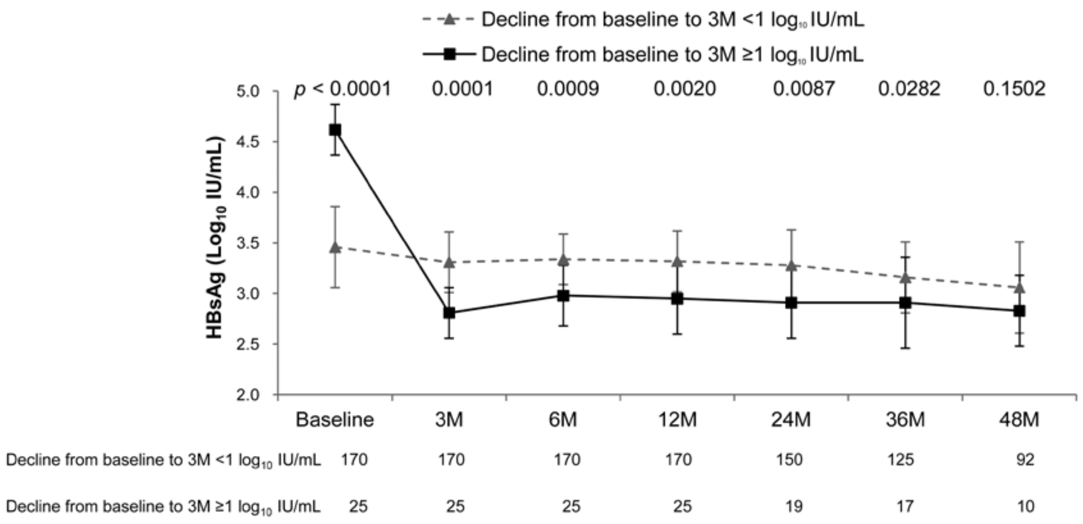
Decline from baseline to 3M <1 log10 IU/mL
Decline from baseline to 3M ≥1 log10 IU/mL
p < 0.0001
0.0001
0.0009
0.0020
0.0087
0.0282
0.1502
HBsAg (Log10 IU/mL)
5.0
4.5
4.0
3.5
3.0
2.5
2.0
Baseline
3M
6M
12M
24M
36M
48M
Decline from baseline to 3M <1 log10 IU/mL 170 170 170 170 150 125 92
Decline from baseline to 3M ≥1 log10 IU/mL 25 25 25 25 19 17 10

Figure S3. Kinetics of on-treatment serum HBsAg levels, stratified by various cutoffs for an HBsAg decline from baseline, at Month 12 of treatment in the HBeAg-negative patients (n = 334). (a) 25%, (b) 50%, (c) 0.5 $\log_{10}$ IU/mL, and (d) 1.0 $\log_{10}$ IU/mL. Error bars indicate the interquartile range. M, months.

(a)

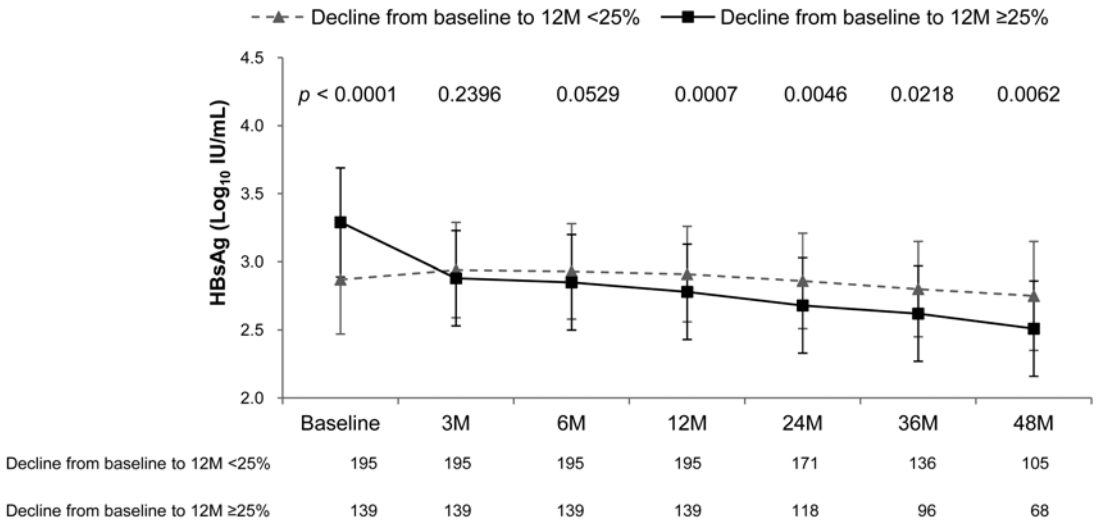

(b)

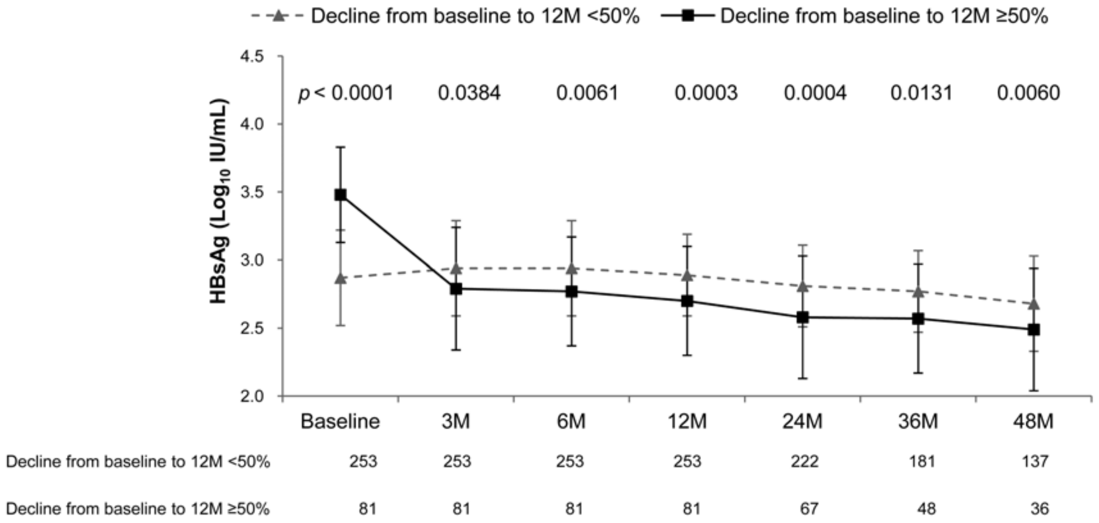

(c)

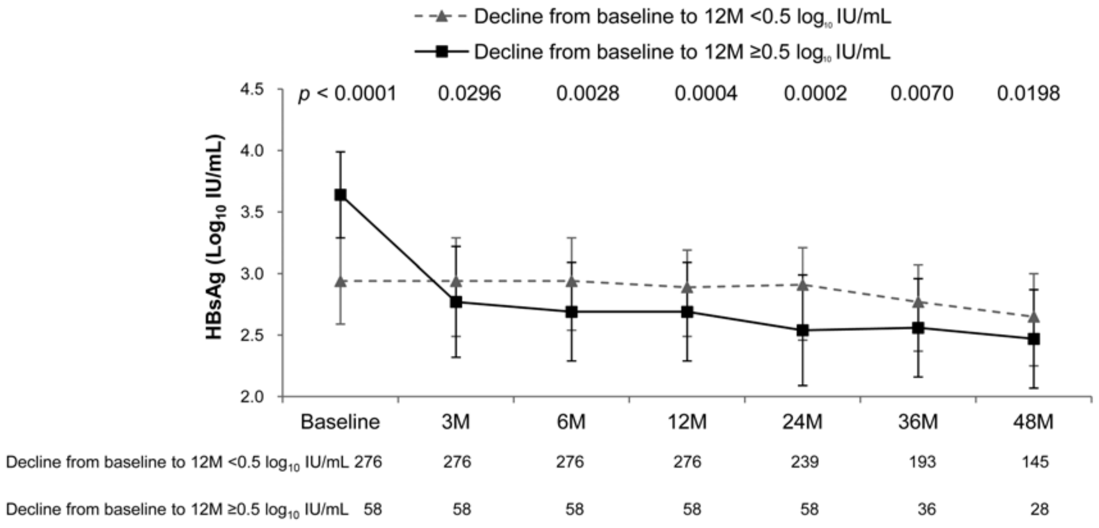
Decline from baseline to 12M <0.5 log10 IU/mL
Decline from baseline to 12M ≥0.5 log10 IU/mL
p < 0.0001
0.0296
0.0028
0.0004
0.0002
0.0070
0.0198
HBsAg (Log10 IU/mL)
4.5
4.0
3.5
3.0
2.5
2.0
Baseline
3M
6M
12M
24M
36M
48M
Decline from baseline to 12M <0.5 log10 IU/mL 276 276 276 276 239 193 145
Decline from baseline to 12M ≥0.5 log10 IU/mL 58 58 58 58 58 36 28

(d)

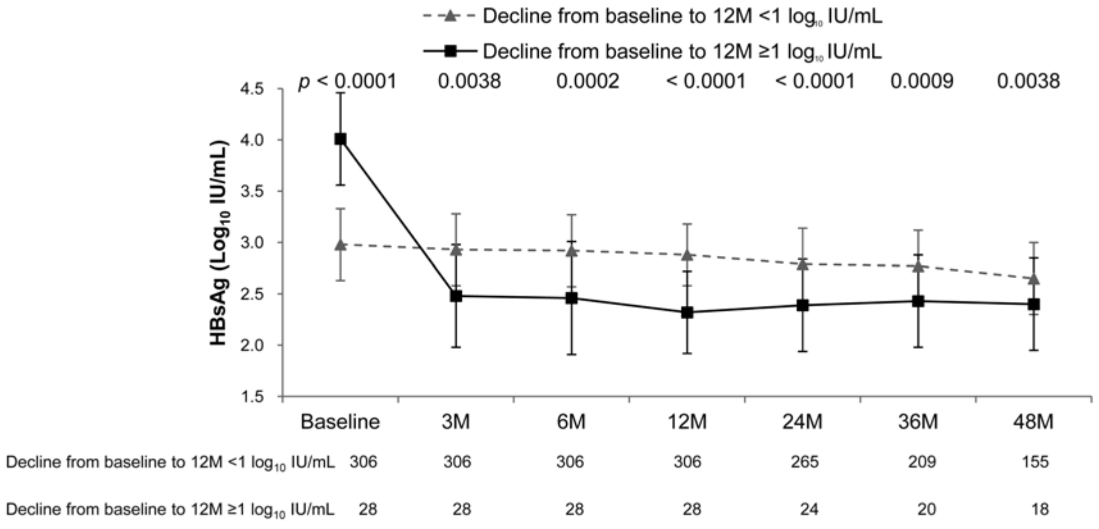
Decline from baseline to 12M <1 log10 IU/mL
Decline from baseline to 12M ≥1 log10 IU/mL
p < 0.0001
0.0038
0.0002
< 0.0001
< 0.0001
0.0009
0.0038
HBsAg (Log10 IU/mL)
4.5
4.0
3.5
3.0
2.5
2.0
1.5
Baseline
3M
6M
12M
24M
36M
48M
Decline from baseline to 12M <1 log10 IU/mL 306 306 306 306 265 209 155
Decline from baseline to 12M ≥1 log10 IU/mL 28 28 28 28 24 20 18

Figure S4. Kinetics of on-treatment serum HBsAg levels, stratified by an HBsAg decline of ≥75% from baseline, at Months 3 or 12 of treatment, or of <75% from baseline, at Month 12 of treatment in the HBeAg-negative patients (n = 334). M, months.

Supplementary Figure 4 HBeAg-negative patients

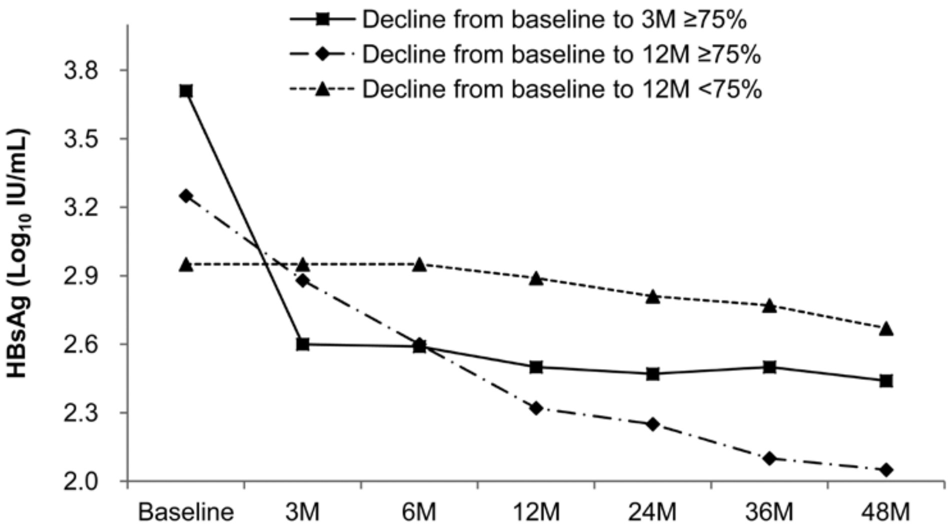

Decline from baseline to 3M ≥75%	43	43	43	43	37	26	21
Decline from baseline to 12M ≥75%	9	9	9	9	7	6	5
Decline from baseline to 12M <75%	282	282	282	282	245	197	147

Figure S5. Kinetics of on-treatment serum HBsAg levels in relation to the achievement of VR. (a) Kinetics of serum HBsAg levels in relation to the achievement of VR during entecavir treatment among HBeAg-positive and -negative patients. (b) Changes in median HBsAg levels during each successive period of treatment. Error bars indicate the interquartile range. VR, virological response.

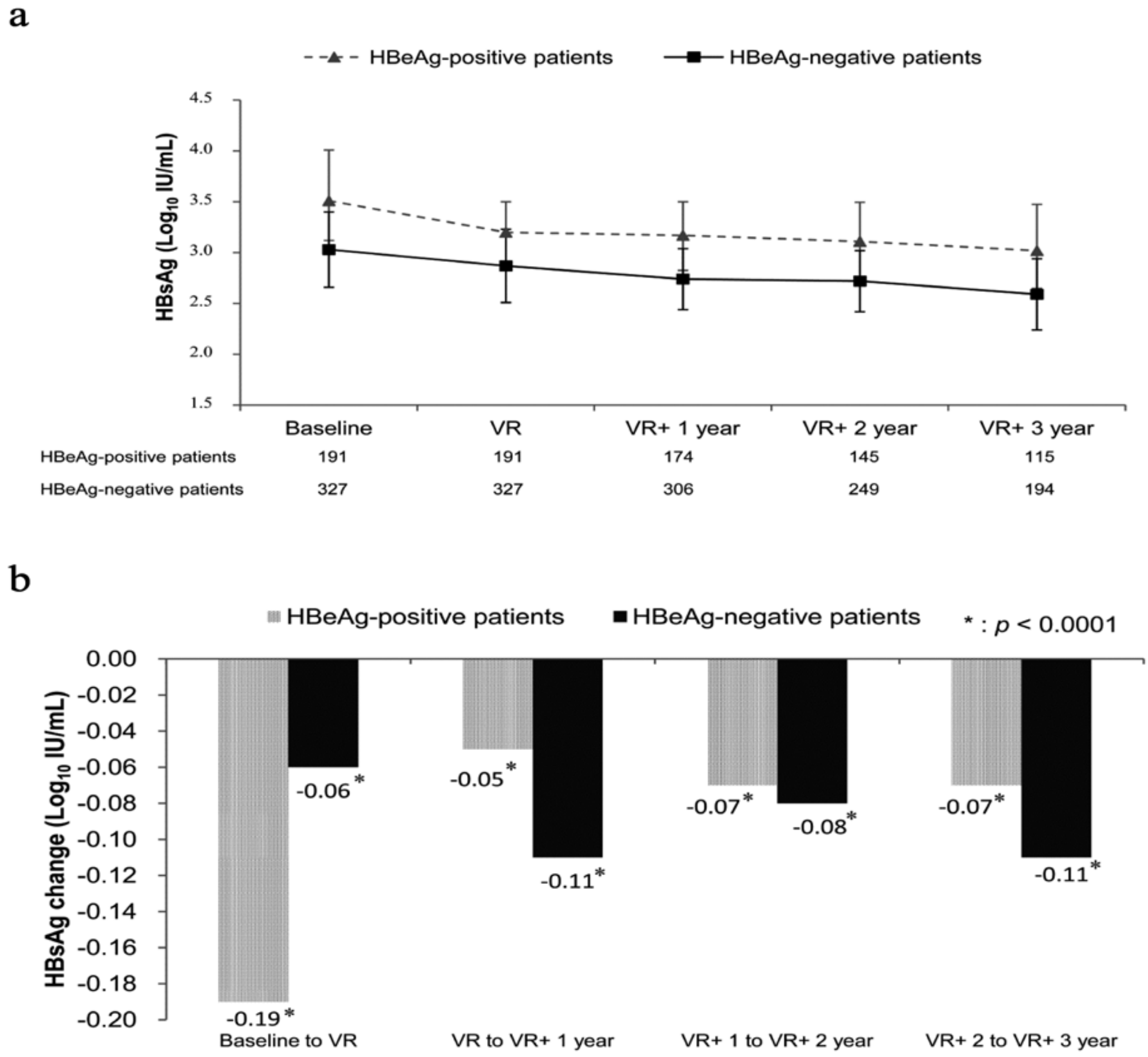

Supplement: Supplementary Information [file srep42879-s1.pdf]
